# Study on the Preparation of Cellulose Acetate Separation Membrane and New Adjusting Method of Pore Size

**DOI:** 10.3390/membranes12010009

**Published:** 2021-12-23

**Authors:** Jianming Wang, Hongchen Song, Longfei Ren, Md Eman Talukder, Shunquan Chen, Jiahui Shao

**Affiliations:** 1 Guangdong Key Laboratory of Membrane Materials and Membrane Separation, Guangzhou Institute of Advanced Technology, Guangzhou 511458, China; jm.wang@giat.ac.cn (J.W.); emant92@gmail.com (M.E.T.); sq.chen@giat.ac.cn (S.C.); 2 School of Environmental Science and Engineering, Shanghai Jiao Tong University, Shanghai 200240, China; longfeiren@sjtu.edu.cn (L.R.); jhshao@sjtu.edu.cn (J.S.)

**Keywords:** cellulose acetate membrane, sponge-like porous structure, pore size control, high-performance separation

## Abstract

As a kind of eco-friendly (biodegradable) material and with a natural anti-fouling ability, cellulose acetate (CA) is more suitable for single-use membrane (especially in bioprocess). In this study, the method for preparing CA membrane by Vapor-assisted Nonsolvent Induced Phase Separation (VNIPS) was studied. The influences of ratio compositions (solid content, acetone/*N*,*N-Dimethylacetamide* ratio, glycerol/CA ratio) and membrane preparation conditions (evaporation time, evaporation temperature and humidity) on the microstructure and other properties were systematically evaluated. Results indicated that acetone/*N*,*N-Dimethylacetamide* ratio and glycerol/CA ratio had great influence on the cross-section structure of membranes. Additionally, the membrane with homogeneous sponge-like porous structure could be prepared stably within certain limits of ratios. Under the premise of keeping the content of other components fixed, the separation membrane with a full sponge pore structure can be obtained when the ratio of glycerol/CA is ≥2.5 or the acetone/solvent ratio is between 0.25 and 0.5. Evaporation time and temperature, humidity and other membrane preparation conditions mainly affected the surface morphology and the pore size. This kind of high-performance membrane with homogeneous sponge-like pore and controllable surface morphology could be potentially used for bioseparation processes.

## 1. Introduction

Cellulose acetate (CA), as a porous membrane material and an important derivative of natural cellulose, has played an important role in membrane separation because of good film forming performance [1,2], high hydrophilicity, easy biodegradation and relatively low cost [3]. At present, the most common method for membrane preparation is phase-inversion method, and the membrane prepared generally present finger-like, sponge-like, or both the two porous structures. Compared with other membrane structures, the all-sponge-like porous structure has a uniform separation layer, narrow pore distribution, stable retention performance, high permeability and high mechanical strength simultaneously, which has become the structural trend of membrane preparation in the future [4]. In addition, the effective pore size is an important index to determine the application of membrane. With the rapid development of pharmaceutical purification, fine chemistry, water treatment and other industries, higher requirements are put forward for the precision separation. It is an important research direction to control the effective pore size of membranes. The pore size of membranes prepared with block copolymers of different chemical composition as material could be precisely regulated within certain range by adjusting the soaking time and temperature in the liquid [5,6,7].

Different from other applications, bioprocesses usually demand single-use membranes, which lead to many single-use membranes needing to be disposed of properly, or they would cause serious pollution. Among all kinds of membrane materials, CA is used to be an important membrane material due to its advantages of wide source, non-toxicity, easy manufacturing technique and degradable properties [8], which is quite suitable for single-use applications. With single-use systems have been treated as future trend in bioprocesses, CA membranes will be given attention again. However, how to regulate the structure of CA membrane effectively and apply to bioseparation are urgent problems that need to be studied [9,10].

CA membranes can prepared by the immersion precipitation method, while isotropic microporous CA membrane without macroporous structure can also be prepared by Thermally Induced Phase Separation (TIPS) [11]. Table 1 shows the performance comparison of different membrane preparation methods. 

The membrane structure and performance are highly demanded in specific applications, and the membrane performance is usually affected by several factors, especially the membrane structure and pore size. Starting from the optimization of the membrane preparation method, the multilevel microstructure of the membrane could be regulated through the control of the membrane forming environment and adjustment of the formula, which has become the main method to get the target membrane. So a new preparation method for CA membrane by VNIPS is worth investigating. In addition, the structure of the CA membrane depends on the composition of casting solution, processing temperature and air relative humidity. The relative humidity is an important parameter among them because it controls the formation of porous or cavities on the surface [12]. Further, the membrane preparation conditions (such as evaporation time, evaporation temperature and relative humidity) were not evaluated systematically in previous studies [13,14,15,16,17].

CA membranes have a wide range of applications in bioprocesses (such as microfiltration, depth filtration, and membrane chromatography). At present, the expensive regenerated cellulose membrane is a main kind of membranes used in bio-separations. However, the literature on the precise control of the biodegradable separation membrane used in the biomedical field is relatively few. This study might provide the reference for promoting the application of cellulose acetate membrane in the precise separation field such as in biomedical purification.

## 2. Materials and Methods

### 2.1. Materials

Cellulose acetate was purchased from Sinopharm Chemical Reagent Co., Ltd.; (Shanghai, China); *N*,*N-Dimethylacetamide* (DMAc, 98%), were obtained from Shanghai Aladdin Biochemical Technology Co., Ltd. (Shanghai, China); glycerin (AR) and acetone (AR) were provided by Sinopharm Chemical Reagent Co., Ltd. (Shanghai, China).

### 2.2. Membrane Fabrication

The casting solution was prepared by blending different compositions of polymers, solvent and additive under constant stirring for 6 h at 25 °C. Acetone/DMAc was used as mixed solvent in the composition. After deaeration, the membranes with thickness of 400μm were scraped on the glass plate (Section 3.1) and supporting layer (Section 3.2) with self-made flat membrane casting machine (FMCM-I). Then CA membranes occurred phase separation through adjusting evaporation temperature, humidity conditions, evaporation time and other factors, which were shown in Table 2. Finally the membranes were immersed in 25 °C water bath to solidify and soaked in pure water for 48 h in order to diffuse the solvent fully.

### 2.3. Characterization of the CA Membranes

#### 2.3.1. Morphology Observation

The morphologies of the CA membranes were observed using scanning electron microscope (SEM, Phenom XL, Phenom-World, Eindhoven, The Netherland). After the freeze drying, the samples were cut off by a knife blade.

#### 2.3.2. Infrared Spectra Measurements (FTIR)

**Figure 1:**
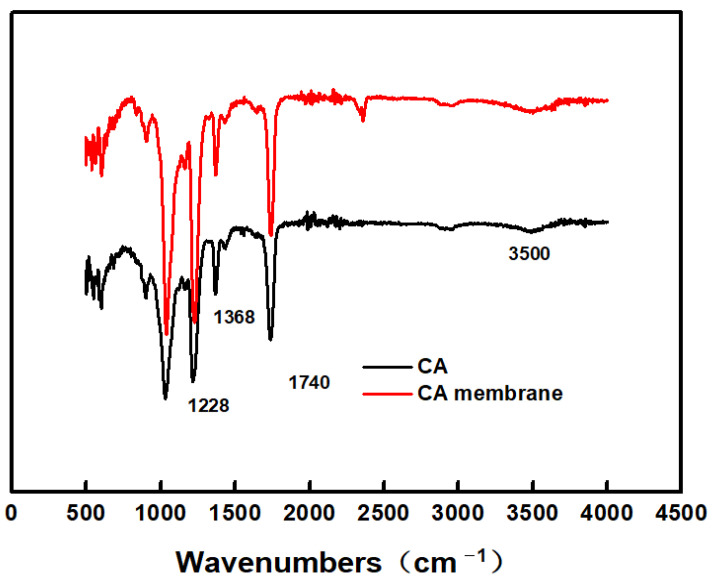
The FT-IR spectra of CA material and CA membrane.

The IR spectra of the cellulose acetate membrane surface were recorded by a spectrometer (FT/IR-6200, Tokyo, Japan) in the range of 4000–500 cm^−1^ and a 4 cm^−1^ resolution. The infrared spectra are illustrated in Figure 1, there is no essential difference in infrared spectra between cellulose acetate material and cellulose acetate film. Several characteristic peaks of CA material and CA membrane were observed, including those at 3500 cm^−1^ (hydroxyl group), 1740 cm^−1^ (carbonyl group), 1368 cm^−1^ (methyl group), and 1228 cm^−1^ (ether group). The above test also shows that there is basically no residual of the solvent and additive in the separation membrane after the sufficient exchange of solvent and non-solvent.

#### 2.3.3. Permeation Characterization

The pure water fluxes of the membranes were determined by a ultrafiltration cell (Model 8010; Millipore). Each membrane was flushed with approximately 100 L/m^2^ of deionized water in advance to remove any agents, then tested under the pressure of 0.10 Mpa. Four samples were measured and the average value is reported. The water flux was calculated using the following equation:

(1) $$J_{v}=\frac{V}{A\cdot t}$$

where *J* is the water flux (L·m^−2^·h^−1^·bar^−1^, LMH·bar^−1^), *V* is the quantity of the permeate (L), *A* is the effective filtration area (m^2^), and *t* is the filtration time (h).

#### 2.3.4. Pore Sizes Analysis

The pore size of the separation membrane was tested by bubble point method. CA membrane was immersed in pure water first no less than 2 h, then put into the bubble point test device, which was connected with the high-purity nitrogen cylinder. The temperature of the test solution was controlled to be 25 ± 1 °C. The pressure was increased slowly until the first bubble appeared on the surface of the membrane. Then the pressure was immediately stopped and the pressure value at this time was recorded as the bubble point pressure. Three readings were taken in order to give an average value. The maximum pore size of the membrane can be calculated according to the following equation:

(2) $$D=4\sigma \;\frac{cos\theta }{\Delta P}$$

where *D* is the pore size (µm), *σ* is surface tension of water (N·m^−1^), *θ* is contact angle between water and membrane (°), Δ*P* is the bubble point pressure (MPa).

#### 2.3.5. Porosity Analysis

Porosity was determined by weighing method. The droplet on the membrane surface was removed first, and the wet weight was weighed on the analytical balance. Then wet membranes were quickly put into the electro-thermostatic blast oven, dried for 3 h at 114 ± 2 °C, and put into the dryer. The dry weight was weighed after cooling. The membrane thickness was measured by thickness gauge (CH-1-S, Shanghai Liuling instrument Co., Ltd., Shanghai, China). For all experiments the average from three replicate tests is reported. The porosity was calculated according to the following equation:

(3) $$\varepsilon =\frac{\left(W_{1}-W_{2}\right)/\rho_{H_{2}O}}{\pi {\left(\frac{d}{2}\right)}^{2}\cdot h}$$

where *W*_1_ and *W*_2_ are the weight of wet membrane and dry membrane respectively, (g); is the density of water, (g·mL^−1^); *d* is the diameter of wet membrane, (cm); *h* is thickness of wet membrane, (cm).

#### 2.3.6. Viscosity Test

The viscosity of the casting solution is a key index that affects the film forming properties, the viscosity was tested by a rotating viscometer at 25 °C (NDJ-8S, Shanghai Pingxuan scientific instrument Co., Ltd., Shanghai, China).

## 3. Results

### 3.1. Effect of Compositions of Casting Solution on Membrane Properties

#### 3.1.1. Effect of CA Content on Membrane Structure and Performance

**Figure 2:**
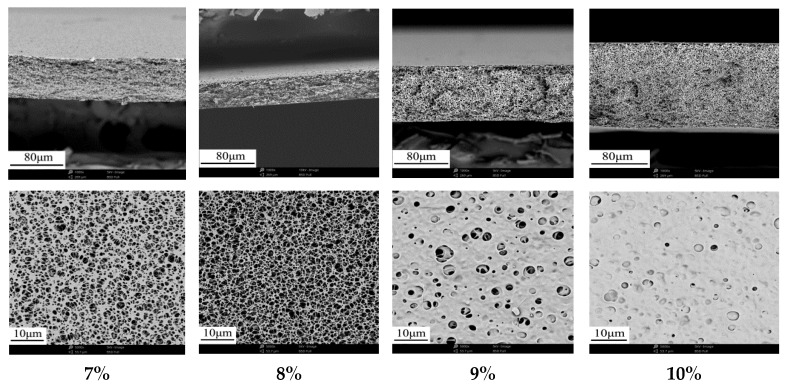
Effect of CA content on the cross-section and surface microstructure of membranes.

Figure 2 shows the effect of solid content on membrane structure. With the increase of solid content, the sponge-like porous structure became denser. The separation membrane with sponge-like porous structure could be prepared stably at certain evaporation temperature (55 °C) and high humidity (≥90%RH). Table 3 shows the effect of solid content on membrane performance. With the increase of solid content, the pore size and porosity of membranes decreased, and the pure water flux decreased rapidly. When CA content was 10 wt%, the maximum pore sizes and porosity were 0.79 μm and 87.5% respectively, and the water flux was only 181.5 L·m^−2^·h^−1^·bar^−1^. This is because the viscosity of casting solution and polymer intermolecular forces increase with the increase of CA content, which lead to the decrease of the moving space between polymer molecules, the slowdown of double diffusion rate between solvent and non-solvent, and the decrease of liquid-liquid phase separation rate. The open porosity of membrane surface was also reduced significantly with the denser sponge-like porous structure in cross-section.

#### 3.1.2. Effect of Acetone/Solvent Ratio on Membrane Structure and Performance

**Figure 3:**
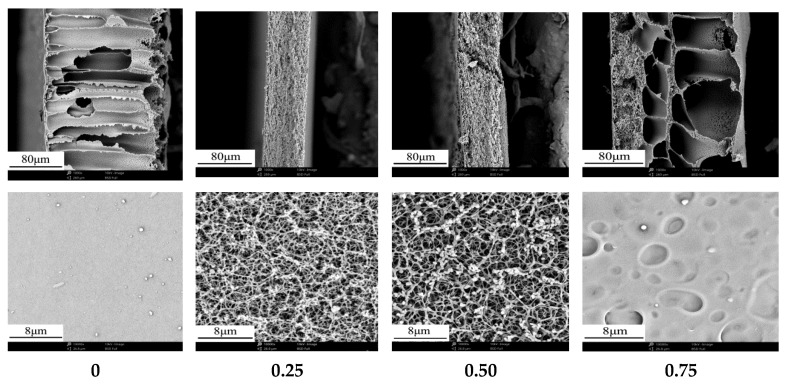
Effect of acetone/solvent ratio on the cross-section and surface microstructure of membranes.

Figure 3 shows the effect of acetone/solvent ratio on membrane structure. As the increase of acetone/solvent ratio, the cross-section structure of membranes changed from finger-like porous structure to sponge-like porous structure, and the membrane surface transformed from dense layer into homogeneous porous layer. When acetone/solvent ratio was 0.75, the cross-section structure of membrane presented the sponge-like porous structure with large pores, but the open porosity of membrane surface was very low. Table 4 shows that the effect of acetone/solvent ratio on membrane performance. With the increase of acetone/solvent ratio, the membrane porosity decreased, meanwhile, the membrane flux and the maximum pore size increased firstly and then decreased. On the one hand, when the membrane was prepared without any acetone in the casting solution, the dense surface layer and finger-like porous structure were formed due to instantaneous phase separation in the water bath. On the other hand, when the membrane was prepared with certain acetone/solvent ratio, the polymer solution presented a stretch state, and formed a large number of polymer networks and small micelle aggregates, which caused the sponge-like porous structure in cross-section and uniform pore size on the surface. However, if acetone content was too high, the membrane preparation system was dominated by volatilize induced phase separation (VIPS), and the network in the polymer micelle aggregations collapsed partially with the rapid volatilization of acetone. The separation membrane contracted seriously, the pore connectivity of the separation membrane pore decreased, and the membrane surface formed a nearly dense structure, which led to the decrease of membrane flux and porosity, and the possibility of defect formation in the membrane.

#### 3.1.3. Effect of Glycerol/CA Ratio on Membrane Structure and Performance

**Figure 4:**
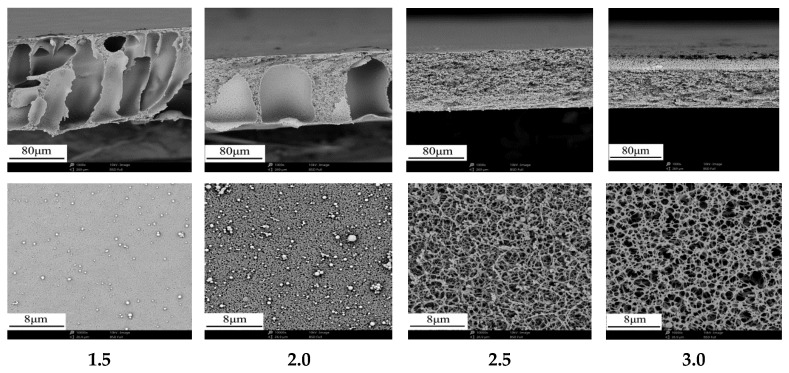
Effects of glycerol/CA ratio on the cross-section and surface microstructure of membranes.

Figure 4 shows the effect of glycerol/CA ratio on membrane structure. With the increase of glycerol/CA ratio, the finger-like porous structure gradually coalesced into macroporous structure and then transformed into homogeneous sponge-like porous structure, which indicated that the cross-section structure of membranes changed from asymmetric structure to symmetrical structure. This structural change is mainly caused by the increase of glycerin content, which improves the viscosity of the system to a certain extent and reduces the phase separation speed. In addition, as a non-solvent, the addition of glycerin changes the stability of the system, which is more conducive to the formation of spongy structure separation membrane. Meanwhile, the membrane surface changed from dense layer to homogeneous porous layer. Table 5 shows the effect of glycerol/CA ratio on membrane performance. It is interesting that the membrane flux increased tenfold approximately with the decrease of maximum pore size. Especially when glycerol/CA ratio was greater than 2.0, the membrane flux increased dramatically. Although the addition of glycerol increased the viscosity of casting solution to a certain extent, the effects on the movement and uniform spatial distribution of molecular fragments were more obvious due to glycerol’s dual function of pore-forming and plasticizing, which resulted in the preparation of a homogeneous sponge-like porous structure separation membrane with high porosity.

### 3.2. Effect of Treatment Conditions on Membrane Properties

#### 3.2.1. Effect of Evaporation Temperature on Membrane Structure and Performance

**Figure 5:**
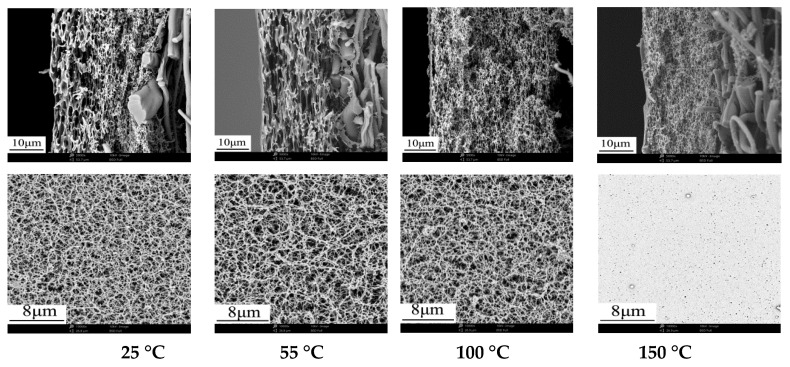
Effect of evaporation temperature on the cross-section and surface microstructure of membranes.

Figure 5 shows the slight effect of evaporation temperature on membrane structure, except for the porous structure of membrane surface when the evaporation temperature is 150 ℃. Table 6 shows the effect of evaporation temperature on membrane performance. As the increase of evaporation temperature, the membrane flux decreased gradually, and the maximum pore decreased from 1.92 μm to 0.81 μm. The separation phase of membranes was determined by the amount of acetone evaporation and water vapor inhalation in VIPS. Additionally, the evaporation temperature could mainly change acetone evaporation situation and control the pore size of the membrane. The higher the temperature was, the faster the acetone evaporated. On the one hand, the escape of volatile solvent resulted in the rapid phase separation and solidification of the casting solution. On the other hand, the membranes showed higher shrinkage as the increase of evaporation temperature, which resulted in the formation of denser porous structure in cross-section and membrane surface. That is why the porous structure of membrane surface was different from other membranes when the evaporation temperature is 150 °C.

#### 3.2.2. Effect of Evaporation Time on Membrane Structure and Performance

**Figure 6:**
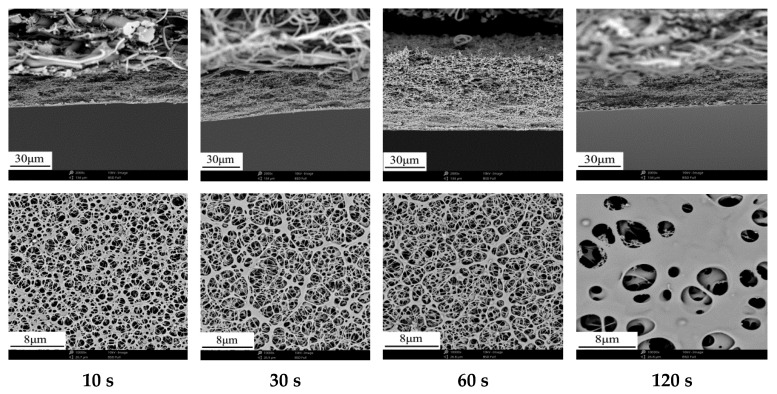
Effect of evaporation time on the cross-section and surface microstructure of membranes.

As mentioned in Section 3.2.1, evaporation temperature that is too high is not suitable for membrane preparation, and the evaporation time could be adjusted instead. Figure 6 shows the effect of evaporation time on membrane structure. With the increase of evaporation time, the open porosity of membrane surface was decreased. The porous structure of membrane surface gradually coalesced into macroporous structure with low porosity, especially when the evaporation time was 120 s, which caused the permeability of membranes were reduced to some extent. Table 7 shows the effect of evaporation time on membrane performance. With the increase of evaporation time, the maximum pore size gradually decreased from 1.70 μm to 1.03 μm, corresponding with the decrease of the water flux. The amount of acetone evaporation and water vapor inhalation could be adjusted by the evaporation time. The longer evaporation time was, the more the acetone evaporated. Therefore, it was easy to form denser porous structure in cross-section.

#### 3.2.3. Effect of Ambient Humidity on Membrane Structure and Performance

**Figure 7:**
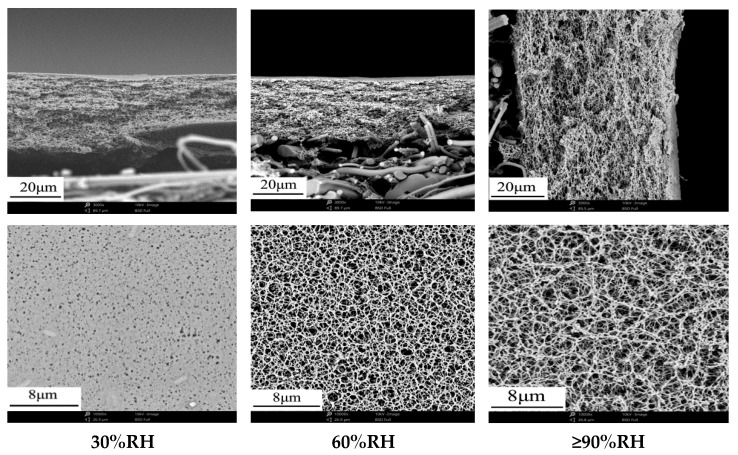
Effect of ambient humidity on the cross-section and surface microstructure of membranes.

Except for the evaporation conditions, ambient humidity also has a noticeable effect on membrane preparation. Figure 7 shows the effect of ambient humidity on membrane structure. With the increase of ambient humidity, the open porosity of membrane surface was increased significantly, which is also the reason for the rapid increase of separation membrane flux. Higher ambient humidity could delay the shrinkage of membrane due to the evaporation of acetone, especially on the membrane surface. Furthermore, the more open porosity of membrane surface could accelerate the evaporation of acetone, which was conducive to the formation of small pore size in cross-section (similar with Section 3.2.1). Table 8 shows the effect of ambient humidity on membrane performance. Higher ambient humidity not only decreased the pore size in cross-section, but also increased the permeability, which could be used to prepare some kinds of high performance membrane.

## 4. Conclusions

Cellulose acetate membranes were prepared by VNIPS in this study. Although the methods of membrane characterization are conventional, the preparation method is creative. The cellulose acetate separation membrane with full sponge-like porous structure could be prepared stably, and the porous structure of membrane surface and the pore size of membranes could be precisely controlled by adjusting the ratio of compositions and the preparation conditions (evaporation temperature, evaporation time and ambient humidity). The following conclusions can be drawn from this study.

The solid content can give an effect on permeability by orders of magnitude; the proper ratios of acetone/solvent and glycerol/CA can not only be contributed to the formation of sponge-like porous structure in cross-section, but also increase the permeability significantly; under the premise of the sponge-like porous structure in cross-section, the preparation conditions (evaporation temperature, evaporation time, and ambient humidity) have greater effect on the open porosity of membrane surface compared with the porous structure in cross-section, which can also adjust the pore size precisely. It can give some references for the preparation of high-performance cellulose acetate separation membranes. How to choose a new and more environmentally friendly volatile solvent system and build a stable and more accurate separation membrane pore control technology are the main challenges at present, and the functional modification of cellulose acetate membrane and its application in the biological field are the main direction of future research.

## Figures and Tables

**Table 1:** Performance comparison of different membrane preparation methods.

Preparation Methods	Technical Features	Membrane Characteristics
Non-solvent induced phase separation (NIPS)	Simple equipment requirements; No high temperature and high pressure; Multiple influencing factors	Dense layer and macroporous structure; low mechanical strength
The thermally induced phase separation (TIPS)	High equipment requirements; High energy consumption	High porosity; high flux; good mechanical strength
Volatilize induced phase separation (VIPS)	Normal temperature and pressure conditions; High humidity and clean steam displacement	Porous membrane structure, large flux; easy to control surface structure
Vapor-assisted nonsolvent induced phase separation (VNIPS)	Composite technology; Specific humidity conditions	Uniform and controllable separation of membrane holes; high porosity; high flux

**Table 2:** Preparation conditions of separation membrane.

NO	Temperature (°C)	Humidity (%)	Evaporation Time (S)	Bath Temperature (°C)
a	25.0	≥90	120	25.0
b	55.0	≥90	120	25.0
c	100.0	≥90	120	25.0
d	150.0	≥90	120	25.0
e	100.0	≥90	10	25.0
f	100.0	≥90	30	25.0
g	100.0	≥90	60	25.0
h	100.0	≥90	120	25.0
i	55.0	30	120	25.0
j	55.0	60	120	25.0
k	55.0	≥90	120	25.0

**Table 3:** The properties of membrane prepared under different CA content conditions.

NO	CA%	Flux (L·m^−2^·h^−1^·bar^−1^)	Bubble Point Pressure (MPa)	Maximum Pore Size (μm)	Porosity (%)	Viscosity (mPa·s)
1	7.0%	14,274.1 ± 485.1	0.154 ± 0.004	1.87	94.7 ± 3.2	750
2	8.0%	8942.9 ± 234.5	0.225 ± 0.006	1.28	92.7 ± 2.5	1310
3	9.0%	1438.5 ± 43.1	0.314 ± 0.010	0.92	88.2 ± 4.5	2410
4	10.0%	181.5 ± 10.2	0.364 ± 0.015	0.79	87.5 ± 5.2	3530

**Table 4:** The properties of membrane prepared under different acetone/solvent ratios.

NO	Acetone/DMAc	Flux (L·m^−2^·h^−1^·bar^−1^)	Bubble Point Pressure (MPa)	Maximum Pore Size (μm)	Porosity (%)	Viscosity (mPa·s)
5	0	4320.0 ± 34.5	0.270 ± 0.010	1.07	93.0 ± 3.1	2415.5
6	0.25	13,012.7 ± 435.1	0.185 ± 0.004	1.56	92.2 ± 1.3	1447.5
7	0.50	13,671.2 ± 345.2	0.215 ± 0.009	1.34	91.7 ± 2.0	1250.5
8	0.75	431.7 ± 10.2	0.203 ± 0.006	1.42	85.4 ± 0.6	987.5

**Table 5:** The properties of membrane prepared under different glycerol/CA ratios.

NO	Glycerol/CA	Flux (L·m^−2^·h^−1^·bar^−1^)	Bubble Point Pressure (MPa)	Maximum Pore Size (μm)	Porosity (%)	Viscosity (mPa·s)
9	1.5	2383.6 ± 25.6	0.124 ± 0.002	2.33	87.5 ± 2.1	947.5
10	2.0	4013.4 ± 120.2	0.265 ± 0.001	1.09	88.8 ± 2.0	1062.0
11	2.5	10,063.6 ± 320.1	0.450 ± 0.005	0.64	91.4 ± 1.5	1174.8
12	3.0	24,472.7 ± 550.5	0.470 ± 0.010	0.61	93.2 ± 1.8	1207.5

**Table 6:** The properties of membrane prepared under different evaporation temperature.

NO	Vaporization Temperature (°C)	Flux (L·m^−2^·h^−1^·bar^−1^)	Bubble Point Pressure (MPa)	Maximum Pore Size (μm)
a	25	5681.9 ± 230.1	0.150 ± 0.002	1.92
b	55	3386.1 ± 160.5	0.160 ± 0.007	1.80
c	100	3036.4 ± 75.9	0.245 ± 0.010	1.18
d	150	2602.7 ± 60.6	0.355 ± 0.015	0.81

**Table 7:** The properties of membrane prepared under different evaporation time.

NO	Vaporization Time (S)	Flux (L·m^−2^·h^−1^·bar^−1^)	Bubble Point Pressure (MPa)	Maximum Pore Size (μm)
e	10	6394.4 ± 235.1	0.170 ± 0.006	1.70
f	30	6341.7 ± 205.2	0.240 ± 0.005	1.20
g	60	3366.6 ± 78.2	0.260 ± 0.008	1.11
h	120	2196.6 ± 50.6	0.280 ± 0.012	1.03

**Table 8:** The properties of membrane prepared under different ambient humidity.

NO	Humidity (%)	Flux (L·m^−2^·h^−1^·bar^−1^)	Bubble Point Pressure (MPa)	Maximum Pore Size (μm)
i	30	4423.2 ± 178.2	0.110 ± 0.002	2.62
j	60	10,248.3 ± 325.2	0.145 ± 0.005	1.99
k	≥90	12,322.7 ± 480.5	0.285 ± 0.012	1.01

## Data Availability

Data sharing not applicable.
